# Tumor microenvironment and immune-related myositis: addressing muscle wasting in cancer immunotherapy

**DOI:** 10.3389/fimmu.2025.1580108

**Published:** 2025-05-02

**Authors:** Shuang Ma, Guangyu Zhao, Shang Sui, Xiankai Chen, Linxin Wu, Taihang Wang, Wanying Xu, Zhijiao Lu, Andong Wang, Xiaolin Wu, Jiaxuan Wu, Yi Liu, Tao Yan

**Affiliations:** ^1^ School of Information Science and Engineering, Shenyang Ligong University, Shenyang, China; ^2^ St. John’s Killmarnock School, Waterloo Region, ON, Canada; ^3^ Department of Thoracic Surgery, National Cancer Center/National Clinical Research Center for Cancer/Cancer Hospital, Chinese Academy of Medical Sciences and Peking Union Medical College, Beijing, China; ^4^ Department of Anesthesiology, National Cancer Center/National Clinical Research Center for Cancer/Cancer Hospital, Chinese Academy of Medical Sciences and Peking Union Medical College, Beijing, China; ^5^ Department of Radiology, Sun Yat-sen Memorial Hospital of Sun Yat-sen University, Guangzhou, China; ^6^ School of Mathematics and Statistics, Liaoning University, Shenyang, China; ^7^ Department of Anesthesiology, Shanxi Province Cancer Hospital/Shanxi Hospital Affiliated to Cancer Hospital, Chinese Academy of Medical Sciences/Cancer Hospital Affiliated to Shanxi Medical University, Beijing, China

**Keywords:** cancer immunotherapy, tumor microenvironment, skeletal muscle, inflammatory cytokines, muscle wasting

## Abstract

Cancer immunotherapy, which leverages the immune system to target neoplastic cells, has undergone significant transformation in recent. However, immunotherapy may have negative effects on skeletal muscle function, causing muscle wasting and functional decline in cancer patients. In this study, we review the mechanisms by which immunotherapy influences skeletal muscle, focusing on immune-related myositis, inflammation, and metabolic alterations within the tumor microenvironment (TME). The key methodologies, including biomechanical assessment techniques such as electrical impedance myography and ultrasound imaging, are discussed to provide valuable insights into process that maintain muscle integrity and function in patients receiving immunotherapy. Moreover, the dual effects of immunotherapy on tumor suppression and muscle damage are described, revealing the significance of inflammatory cytokines, immune checkpoints, and metabolic disturbances within the TME. Importantly, we propose combination therapies integrating immunotherapy and nutritional interventions or anti-inflammatory interventions as potential approaches for mitigating muscle wasting. This study highlights the need for deeper investigations to optimize immunotherapy and improve its efficacy in preserving muscle health, thereby improving patient outcomes and quality of life.

## 1 Introduction

Cancer is a leading cause of mortality worldwide and recent advances have led to the discovery of new therapeutic approaches. Although conventional treatments such as surgery, radiotherapy, and chemotherapy can significantly improve survival rates of cancers patients (1), these treatments are often associated with substantial adverse side effects, particularly skeletal muscle dysfunction. In recent years, new discoveries in the field of immunotherapy have revolutionized cancer treatment strategies (2). By harnessing the patient’s immune system to enhance tumor cell recognition and elimination, immunotherapy offers possess significant potential to treat complex diseases, but immune-related adverse events need to be addressed. For instance, patients undergoing immunotherapy often present with pronounced alterations in skeletal muscle function (3), such as immune-related myositis and, in some cases, myasthenia gravis. Although immunotherapy may cause some skeletal muscle damage, it has the following significant advantages over traditional therapies: a. Lower cytotoxicity: Immunotherapy mainly attacks tumor cells by activating or regulating the immune system rather than directly damaging cells, so it causes less direct damage to normal tissues and has lower cytotoxicity. b. Persistent immune memory: Immunotherapy can induce persistent immune memory, allowing patients to maintain long-term immune surveillance of tumors after treatment, which is not possible with traditional therapies. c. Better specificity: Immunotherapy generally has higher specificity and can more accurately identify and attack tumor cells, thereby reducing the impact on normal cells (4). Among these, PD-1/PD-L1 inhibitor therapy and CAR-T cell therapy will be the focus of this review. In light of the diverse immune-related adverse events associated with immunotherapy, this review specifically concentrates on immune-related myositis. Myositis represents one of the more prevalent and clinically significant muscle-related adverse events, characterized by inflammation and necrosis of muscle fibers, which culminates in muscle weakness and functional decline (5). Although other immune-related neuromuscular complications, such as myasthenia gravis, are also of considerable importance, myositis has been selected as the primary focus due to its higher incidence and its direct impact on muscle integrity and patient quality of life. A comprehensive understanding of the mechanisms and clinical manifestations of myositis is essential for the development of effective management strategies aimed at mitigating muscle toxicity and enhancing patient outcomes (6). Skeletal muscle functions as the principal effector organ for locomotor activity and plays a critical role in metabolic and immune regulation (7). Cancer progression and prolonged treatment often lead to muscle impairment, cachexia, and functional decline (8, 9). These advances have not only significantly improved patients’ survival rates and quality of life, but also opened up new avenues for cancer treatment.

Cytotoxic pharmacological interventions, which are crucial to cancer treatment, induce dual-spectrum myocyte perturbations (both immediate and secondary) during neoplastic targeting (10). Contemporary investigations have shown that chemoimmunotherapeutic regimens not only elicit oxidative stress-mediated impairment of myocyte bioenergetic capacity but also accelerate myofibrillar catabolism by inducing pro-inflammatory cytokine cascades (11). Tumor environment (TME) is a major factor influencing cancer therapy (12). The TME actively facilitates tumor growth, metastasis, immune evasion, drug resistance, and therapeutic responsiveness and is defined as the complex milieu comprising non-neoplastic cells, extracellular matrix components, and signaling molecules surrounding tumor cells (12). Immunotherapy modulates the TME by altering the balance between immunostimulatory and immunosuppressive cells, thereby amplifying anti-tumor immunity (13). However, these modalities are associated with immune-mediated adverse events, particularly immunogenic myopathies, characterized by interleukin-driven multisystem cytokine dysregulation that propagates myocellular damage, culminating in the diminution of both oncological response metrics and patient-reported outcome measures (14). This pathophysiological interplay necessitates further investigations into immunotherapy-associated myocellular remodeling mechanisms (15), particularly the TME crosstalk dynamics, to develop musculoskeletal interventions that concurrently improve patient treatment while also ameliorating cancer survivorship trajectories (16). Therefore, it is particularly important to deeply explore the mechanism of the impact of immunotherapy on skeletal muscle function.

In recent year, several studies are increasingly studying the iatrogenic potential of immunotherapeutic modalities (17, 18), particularly their propensity to elicit myopathic sequelae through complex pathophysiological mechanisms (19). Researchers have directed their efforts to develop cytoprotective interventions, including immunoregulatory cell modulation, metabolic network reprogramming, and precision dosing that improve the musculoskeletal toxicity profiles of immuno-oncological agents (13). The iatrogenic manifestations of such patients extend beyond mere sarcopenic alterations, to include occurrence of systemic immunotolerance that undermines anti-tumor surveillance (20). Consequently, it is crucial to explore the bidirectional crosstalk between immunotherapeutic agents, the TMEal niche, and clarify their collective effect on myocellular homeostasis to improve the prognosis of patients (21).

**Figure 1:**
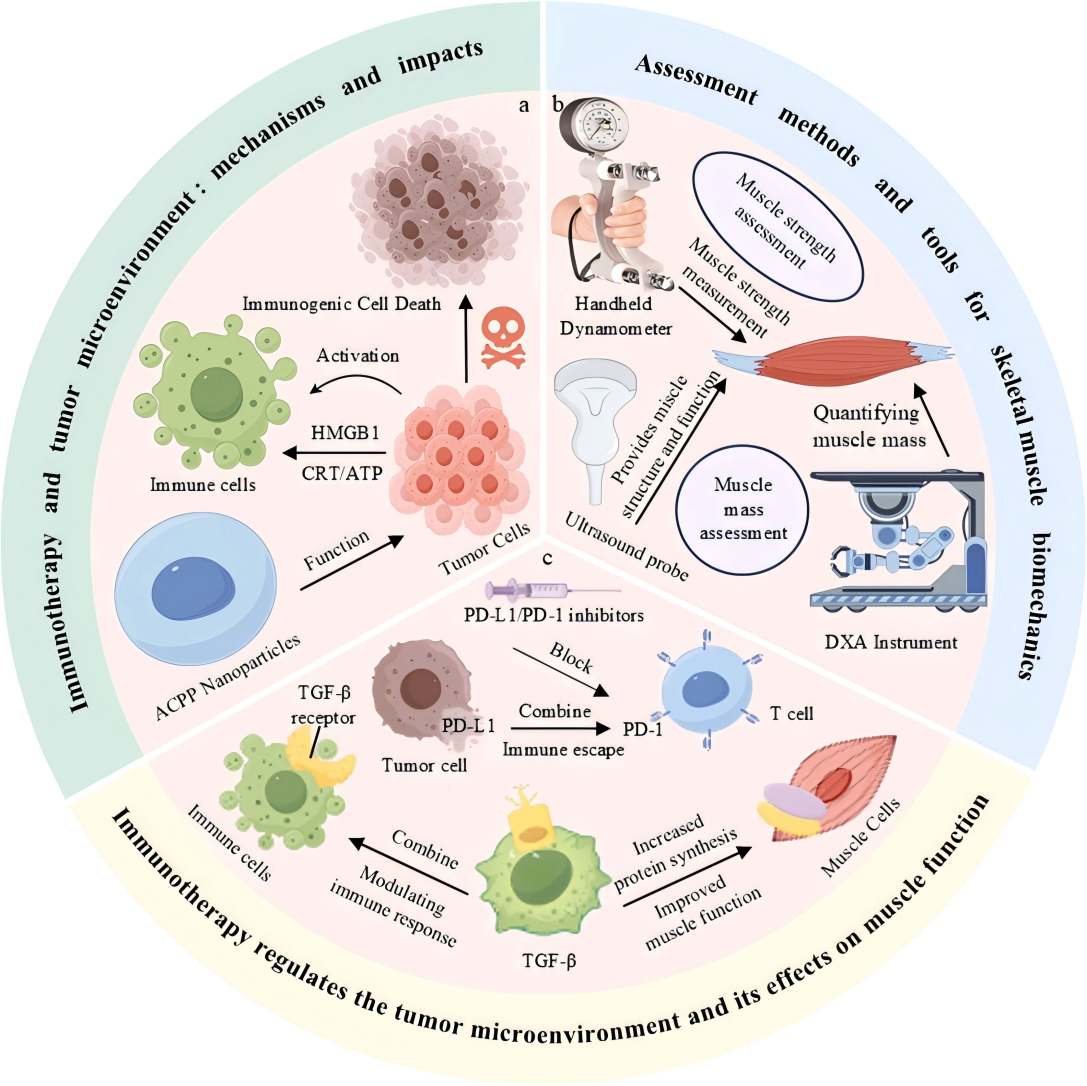
Immunotherapy modulates the tumor microenvironment and influences skeletal muscle function. **(a)** Immune activation and tumor cell death mediated by immunogenic signals. **(b)** Common tools for skeletal muscle strength and mass assessment. **(c)** Immunotherapeutic blockade of PD-1/PD-L1 and TGF-β pathways enhances immune response and improves muscle function

In this review, we systematically examined the effects of immunotherapy on skeletal muscle function and its associated mechanisms (**Figure 1**). Initially, we investigated the mechanisms by which immunotherapy triggers immune-related adverse events (e.g.immune-mediated myositis) and systemic inflammatory responses that disrupt skeletal muscle biology and lead to muscle atrophy, cachexia, and functional decline. Subsequently, we evaluated the performance of currently used biomechanical assessment techniques to detect skeletal muscle injury, emphasizing their utility in monitoring immunotherapy-related muscular dysfunction. Finally, we discussed the bidirectional crosstalk between immunotherapy and the TME, particularly how immune checkpoint blockade (e.g., PD-1/PD-L1 inhibition) disrupts tumor immune evasion while causing unintended side effects on skeletal muscle homeostasis. Strategies for enhancing immunotherapy protocols, such as the temporal modulation of dosing, combination with anti-inflammatory agents, and precise targeting of TME components, have been proposed to prevent muscle toxicity while also enhancing anti-tumor efficacy.

## 2 Immunotherapy-mediated modulation of the TME and skeletal muscle function

### 2.1 Regulation of the TME by immunotherapy

In recent years, immunotherapy has emerged as a significant advancement in the treatment of tumors. This therapeutic approach functions by recognizing and targeting tumor cells through the activation or modulation of the patient’s own immune system. Central to this process are the immune checkpoints PD-1 (programmed death receptor-1) and its ligand PD-L1, which play a crucial role in the regulation of T cell immune activity. Tumor cells frequently evade immune detection by overexpressing PD-L1, thereby suppressing T cell proliferation and function. Inhibitors of the PD-1/PD-L1 pathway counteract this evasion by blocking the interaction between PD-1 and PD-L1, thereby restoring T cell activity and enhancing anti-tumor immune responses, which has led to a marked improvement in the survival rates of patients with various malignant tumors (22). Additionally, CTLA-4 (cytotoxic T lymphocyte-associated protein 4) represents another pivotal immune checkpoint molecule. Inhibitors targeting CTLA-4 bind to this molecule on the surface of T cells, disrupting its interaction with CD80 and CD86 on antigen-presenting cells. This disruption enhances T cell activation and proliferation, thereby promoting robust anti-tumor immune responses (23). Chimeric antigen receptor T cell (CAR-T) therapy represents a pioneering advancement in cellular therapy. This approach involves the genetic modification of a patient’s T cells to express chimeric antigen receptors (CARs), which are designed to specifically target antigens on the surface of tumor cells, thereby augmenting the T cells’ capacity to identify and eradicate malignant cells. CAR-T cell therapy has demonstrated substantial efficacy in the management of hematological malignancies; however, its application in the treatment of solid tumors is hindered by challenges such as immunosuppression within the TME (24). These immunotherapeutic strategies modulate the immune system through various mechanisms, offering novel prospects and methodologies for cancer treatment.

Recently, immunotherapy has shown great potential to treat cancer, by enhancing anti-tumor immune responses and modulating immune cell function within the TME (2, 19). The TME not only contains tumor cells but also immune cells, stromal cells, vasculature, extracellular matrix, and various signaling molecules (25). The intricate interactions among these components influence the tumor growth, metastasis, and immune evasion (25, 26). Immunotherapy aims to modify these elements to regulate the immune microenvironment, thereby enhancing the ability of the immune system to recognize and eliminate tumor cells (27). For instance, it significantly improves anti-tumor responses by activating immune cells, particularly T cells and natural killer (NK) cells, which enhances cancer treatment efficacy (28). Beside modulating conventional immune checkpoint inhibitors such as PD-1/PD-L1 inhibitors, immunotherapies that target specific immunosuppressive cells within the TME are increasingly recognized as promising therapeutic strategies (29). For instance, polymorphonuclear myeloid-derived suppressor cells (PMN-MDSCs) are involved in the regulation of immune suppression within the TME, facilitating immune evasion by inhibiting anti-tumor functions of immune cells (30). This, in turn, promotes tumor progression and metastasis. Therefore, targeting immunosuppressive cells to neutralize their effects within the TME is proposed as an alternative approach for improving immunotherapy research (31).

Metabolic reprogramming of tumor and immune cells is increasingly studies in recent years (32). Tumor cells often alter their metabolic pathways characterized by increased consumption of glucose, amino acids, and lipids to sustain their proliferation (33). Metabolic reprogramming within the TME not only supports tumor cell survival, growth, and metastasis but also affects immune cell function (34). For example, metabolic alterations in the TME can decrease immune cell activity by disrupting glycolysis in T cells, thereby reducing their anti-tumor capacity and fostering immune tolerance (34, 35). This indicates that modulating these metabolic pathways may be a novel therapeutic approach to enhancing immune responses and improving immunotherapy outcomes.

Long non-coding RNAs (lncRNAs) play major regulatory roles in the TME (36). Research has indicated that they influence immune cell function by modulating immune checkpoint expression, to alter immune cell-mediated anti-tumor activity and tumor progression (36, 37). The expression levels of lncRNAs affect the immune evasion, immunosuppression, and therapeutic responses in cancer (37). Consequently, the specific lncRNAs that modulate anti-tumor immune responses are being studies to provide modalities for patient treatment.

In summary, immunotherapy enhances anti-tumor immune responses by modulating immune cell composition, function, and metabolic characteristics within the TME (38). Therefore, research need to identify molecular targeting approaches to optimize the efficacy of immunotherapy. For instance, integrating immunotherapy with metabolic reprogramming, immunosuppressive cell targeting, and lncRNA regulation can promote the implementation of personalized, precise, and highly effective treatment strategies for patients with cancer.

### 2.2 Mechanistic interplay between immunotherapy and the TME

#### 2.2.1 Immune checkpoint modulation

Immunotherapy significantly enhances anti-tumor immune responses by activating immune cells, inhibiting immune escape pathways, regulating cytokines, and remodeling the immunosuppressive microenvironment (39). However, these mechanisms may also lead to immune-related side effects, especially on skeletal muscle. While attacking tumor cells, T cells and NK cells activated by immunotherapy may mistakenly identify skeletal muscle cells as targets, triggering an inflammatory response (40, 41). In addition, the systemic inflammatory response and cytokine release induced by immunotherapy can aggravate muscle protein breakdown and mitochondrial dysfunction, affecting muscle function (42). Immune checkpoint inhibitors, including PD-1/PD-L1 and CTLA-4 inhibitors, have demonstrated significant efficacy in restoring immunological function and counteracting tumor immune evasion (43). These inhibitors specifically augment the tumoricidal potential of T cells by disrupting co-inhibitory signaling pathways, thereby achieving sustained clinical remission across various malignancies (43). Nonetheless, therapeutic resistance is prevalent among a substantial subset of patients, attributed to the emergence of immunologically “cold” tumor phenotypes. These phenotypes are characterized by inadequate infiltration of cytotoxic T-lymphocytes and impaired immune synapse formation within the TME (44). Research indicates that patients receiving PD-1 inhibitors may experience a higher incidence of immune-related myositis compared to those undergoing conventional therapies. Additionally, immune checkpoint inhibitors may modulate cellular senescence by facilitating the clearance of senescent cells, thereby diminishing the release of the senescence-associated secretory phenotype (SASP), which can indirectly confer beneficial effects on skeletal muscle function (45).

**Figure 2:**
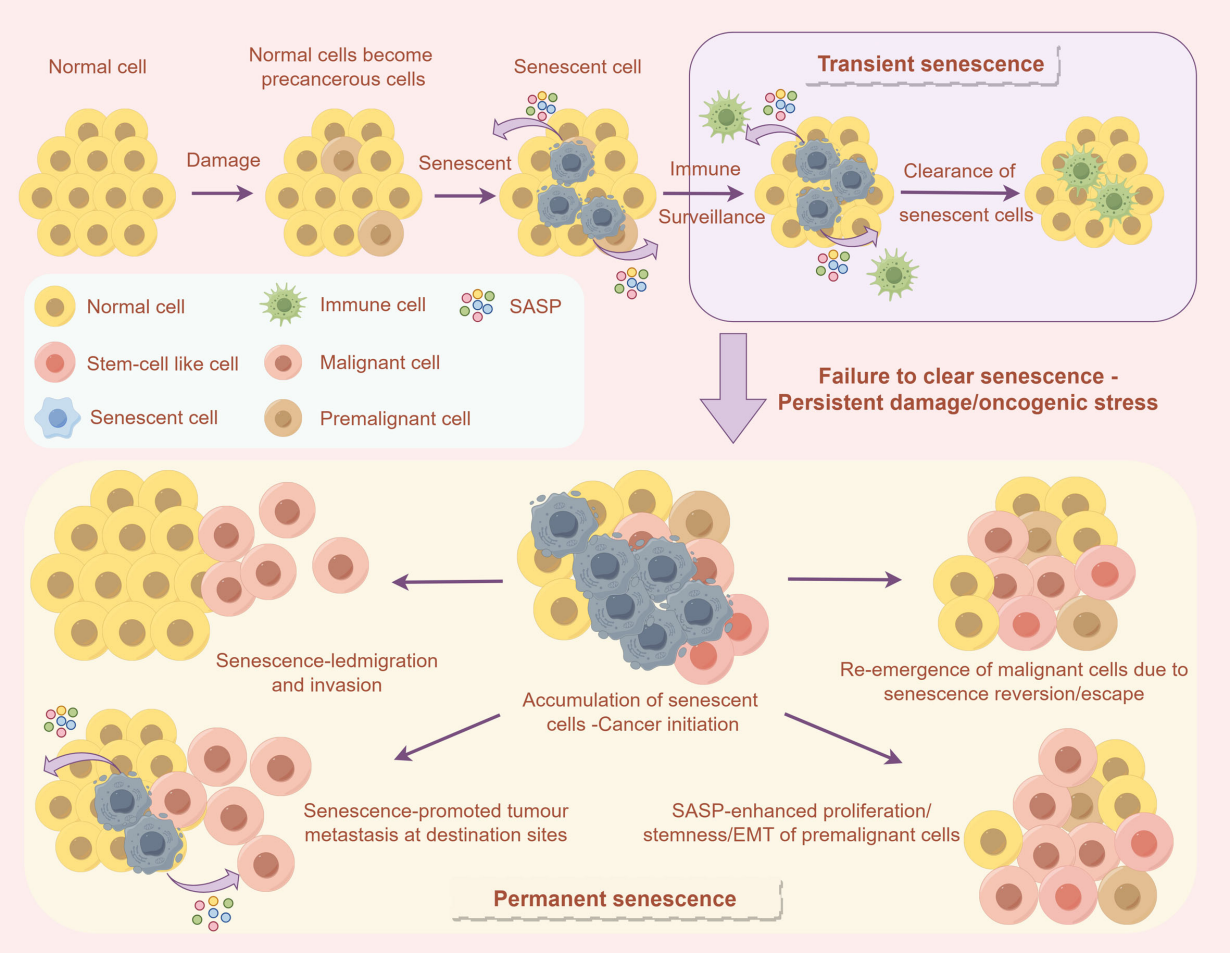
The influence of cellular senescence on the TME and immune response. The upper section describes how normal cells can enter a temporary senescent state following damage, subsequently being removed through immune surveillance. Conversely, the lower section explains that if senescent cells are not efficiently cleared, they may accumulate as a result of sustained damage or oncogenic stress. This accumulation can result in permanent senescence, thereby facilitating tumor initiation, invasion, and metastasis.

As depicted in **Figure 2**, Immunotherapy, while modulating the TME, also influences the process of cellular senescence, thereby exerting an indirect impact on skeletal muscle function (46). Cellular senescence can be categorized into two types: transient and permanent (47, 48). In transient senescence, normal cells enter a senescent state following damage or oncogenic stress (49), during which the immune surveillance mechanism can eliminate these senescent cells, thereby maintaining tissue homeostasis (50). However, immunotherapy may disrupt this process (51). For instance, immune checkpoint inhibitors might indirectly facilitate the clearance of senescent cells and reduce the release of the senescence-associated secretory phenotype (SASP) by enhancing the immune system’s attack on tumor cells, thus mitigating its adverse effects on muscle function (49, 51). Conversely, the inflammatory response induced by immunotherapy may expedite cellular senescence, elevate SASP secretion, and further impair muscle health (52). Consequently, comprehending the dual effects of immunotherapy on cellular senescence is crucial for thoroughly evaluating its impact on skeletal muscle function and for providing a foundation to optimize treatment strategies (53).

#### 2.2.2 Cytokine-driven mechanisms

Immunotherapy-activated T cells and NK cells can mistakenly target skeletal muscle causing inflammation. Cytokines and chemokines have been shown to stimulate muscle cell apoptosis and protein breakdown, while also activating pathways like NF-κB and JAK-STAT to boost the secretion of inflammatory factors, creating a feedback loop that worsens muscle inflammation. Tumor necrosis factor-alpha (TNF-α) activates the NF-κB pathway, increasing muscle protein breakdown, while IFN-γ enhances the immune cell activity via activating the JAK-STAT pathway, which aggravates inflammation (54). The inflammatory response elicited by immunotherapy can activate downstream signaling cascades, notably the NF-κB pathway, which subsequently enhances the expression of proinflammatory cytokines, including TNF-α and IL-6. These cytokines exacerbate muscle protein catabolism and impair the regenerative capacity of muscle stem cells, leading to reduced muscle function (55). Clinical evidence from patients undergoing treatment with immune checkpoint inhibitors indicates that elevated cytokine levels are associated with an increased incidence of muscle weakness and fatigue. Management strategies, such as monitoring inflammatory markers and adjusting treatment regimens, can assist in mitigating these adverse effects (56).

#### 2.2.3 Metabolic alterations

The impact of immunotherapy on skeletal muscle function is complex and involves a sophisticated interaction between inflammatory and metabolic pathways (26). The inflammatory response induced by immunotherapy can activate downstream signaling cascades, such as the NF-κB pathway, which subsequently promotes the expression of proinflammatory cytokines, including TNF-α and IL-6. These cytokines not only intensify muscle protein catabolism but also impair the regenerative capacity of muscle stem cells, resulting in diminished muscle function (4). Metabolic disturbances within the TME further aggravate these effects by disrupting energy homeostasis and impairing contractile performance through the induction of oxidative stress and mitochondrial dysfunction in myofibers (57). Tumor cells frequently modify their metabolic pathways, which is evidenced by an increased uptake of glucose, amino acids, and lipids to support their proliferation (58). This metabolic reprogramming within the TME not only facilitates tumor cell survival, growth, and metastasis but also influences immune cell function. For example, metabolic changes in the TME can impair immune cell activity by disrupting glycolysis in T cells, thereby diminishing their anti-tumor efficacy and promoting immune tolerance (35). Nutritional interventions, such as high-protein diets and omega-3 fatty acids, may mitigate these metabolic effects and promote muscle health. Furthermore, the gut microbiota plays a pivotal role in modulating systemic inflammation and immune responses, presenting novel therapeutic opportunities to maintain muscle health during immunotherapy (59).

### 2.3 Comparison of the effects of immunotherapy and chemotherapy on skeletal muscle

Immunotherapy and chemotherapy represent two principal modalities in cancer treatment, each exerting distinct impacts on skeletal muscle function. Chemotherapeutic agents predominantly influence skeletal muscle via direct cytotoxic effects, culminating in muscle atrophy and diminished functionality (60). For instance, specific chemotherapeutic agents, including cyclophosphamide, doxorubicin, and docetaxel, affect muscle cells through various mechanisms, resulting in muscle weakness, fatigue, and atrophy. These agents may induce muscle protein degradation and mitochondrial dysfunction through pathways involving oxidative stress, inflammatory responses, and direct cellular damage. Immunotherapy augments the anti-tumor response by modulating the immune system; however, it may concurrently induce immune-related adverse effects and impair skeletal muscle function (61). Specifically, immune checkpoint inhibitors, such as PD-1/PD-L1 inhibitors, reinstate the immune system’s capacity to identify tumors by obstructing the pathways utilized by tumor cells to evade immune surveillance. Nonetheless, this method of immune system activation can result in immune-related myositis, thereby affecting muscle function (62). Furthermore, the inflammatory response and metabolic alterations induced by immunotherapy may detrimentally impact skeletal muscle, leading to muscle protein degradation and mitochondrial dysfunction.

In contrast to chemotherapy, the impact of immunotherapy on skeletal muscle is primarily associated with the modulation and activation of the immune system. The engagement of immune cells and the subsequent release of inflammatory mediators can exert direct or indirect effects on the metabolism and structural integrity of muscle cells, thereby influencing muscle function (63). For instance, immunotherapy may alter muscle strength and endurance by elevating proinflammatory cytokine levels and facilitating the catabolism of muscle proteins. Specifically, the administration of immune checkpoint inhibitors can result in the hyperactivation of T cells and NK cells, which may erroneously target skeletal muscle cells, thereby initiating inflammatory responses and causing muscle damage. The impact of immunotherapy on skeletal muscle likely encompasses the modulation of muscle stem cells, such as satellite cells, which are crucial for muscle repair and regeneration following injury (64). Immunotherapy may influence the reparative and regenerative capabilities of muscles by altering the activity of these stem cells. For instance, immune checkpoint inhibitors may modify the microenvironment surrounding muscle stem cells, thereby affecting their proliferation and differentiation, which in turn could influence the restoration of muscle function (65).

Furthermore, the systemic inflammatory response induced by immunotherapy may indirectly impact skeletal muscle. Persistent inflammatory conditions can result in enhanced muscle protein catabolism and suppression of muscle protein synthesis, thereby exacerbating muscle atrophy and functional deterioration (66). This systemic effect may be particularly pronounced in patients undergoing prolonged immunotherapy. In clinical practice, the concurrent use of immunotherapy and chemotherapy necessitates careful consideration of their potential impacts on skeletal muscle function (67). A comprehensive understanding of the distinct mechanisms by which these treatments affect skeletal muscle is crucial for the development of more effective intervention strategies. Such understanding can aid in minimizing muscle toxicity and enhancing both the quality of life and treatment tolerance for patients (68). Future research should focus on investigating the long-term effects of immunotherapy and chemotherapy on skeletal muscle, as well as on formulating protective measures to optimize integrated treatment regimens.

In summary, immunotherapy and chemotherapy exert distinct mechanisms of action on skeletal muscle function, yet both can result in a deterioration of muscle performance. In clinical practice, it is imperative to thoroughly consider these effects. By designing and monitoring treatment plans judiciously, it is possible to mitigate damage to skeletal muscle, thereby enhancing therapeutic outcomes and improving patients’ quality of life (69).

### 2.4 Effects of immunotherapy on muscle function: clinical evidence and mechanism discussion

Immunotherapy has achieved substantial advancements in the treatment of tumors; however, its impact on muscle function has increasingly garnered scholarly attention. Clinical studies and case reports offer valuable insights into immunotherapy-induced muscle dysfunction and elucidate its potential mechanisms (70). In recent years, numerous clinical investigations have examined the effects of immunotherapy on muscle function. For instance, a retrospective study involving patients with non-small cell lung cancer found that approximately 15% of patients experienced varying degrees of muscle weakness and fatigue following treatment with PD-1 inhibitors, with 5% exhibiting more severe symptoms that impaired their daily activities (71). Additionally, a prospective cohort study reported that around 20% of melanoma patients receiving CTLA-4 inhibitors developed immune-related myositis, with 10% necessitating the suspension or discontinuation of treatment. In a clinical trial involving patients with renal cell carcinoma, approximately 12% of those treated with PD-1/PD-L1 inhibitors experienced symptoms of muscle weakness and fatigue, with 3% necessitating hospitalization (72). These findings indicate that immunotherapy-induced muscle dysfunction presents with a notable incidence and severity in clinical settings.

In clinical practice, there is a growing body of evidence documenting cases of muscle dysfunction induced by immunotherapy. For instance, a case series study identified five patients undergoing treatment with PD-1/PD-L1 inhibitors, three of whom exhibited progressive muscle weakness and atrophy, while two developed severe inflammatory myopathy (73). Muscle biopsies from these patients revealed significant inflammatory cell infiltration and necrosis within muscle fibers, suggesting that immunotherapy may directly target muscle tissue through immune system activation. Additionally, a separate case report describes a patient who underwent CAR-T cell therapy and subsequently experienced severe muscle atrophy and dysfunction, accompanied by a systemic inflammatory response (74). The muscle biopsy of this patient demonstrated extensive CD8+ T cell infiltration in muscle fibers, indicating that immunotherapy might directly affect muscle tissue by activating specific immune cells. These case reports underscore the potential risks associated with immunotherapy on muscle function, particularly in the context of long-term treatment.

Muscle dysfunction induced by immunotherapy is characterized by several underlying mechanisms. Firstly, immune checkpoint inhibitors play a crucial role by potentially misidentifying muscle cells as targets, thereby activating T cells and natural killer (NK) cells and initiating an inflammatory response (4). Secondly, the systemic inflammatory response elicited by immunotherapy can contribute to muscle weakness and atrophy through increased muscle protein degradation and mitochondrial dysfunction. Lastly, immunotherapy may exacerbate muscle dysfunction by impairing the activity of muscle stem cells, known as satellite cells, thus inhibiting muscle repair and regeneration (75). These mechanisms align with clinical observations of muscle dysfunction, such as weakness and atrophy, underscoring the multifaceted impact of immunotherapy on muscle function.

Data from clinical studies and case reports demonstrate a strong correlation with the aforementioned mechanisms. For instance, the infiltration of inflammatory cells and the presence of necrosis observed in muscle biopsies align with the effects of immune checkpoint inhibitors in activating T cells. Furthermore, the degradation of muscle proteins and mitochondrial dysfunction are consistent with the mechanisms underlying the systemic inflammatory response. These findings offer a theoretical foundation for the development of intervention strategies aimed at mitigating muscle toxicity associated with immunotherapy.

## 3 Assessment of skeletal muscle biomechanics

### 3.1 Evaluation of muscle strength

Conventional methods for evaluating muscle strength include the use of handheld and hydraulic dynamometers (76). A portable dynamometermeasures muscular strength by quantifying the force generated during maximal voluntary contractions (77). Grip strength assessments are also frequently performed using a hydraulic dynamometer specifically designed to evaluate forearm and hand grip strength, based on the principle of force measurement.

**Figure 3:**
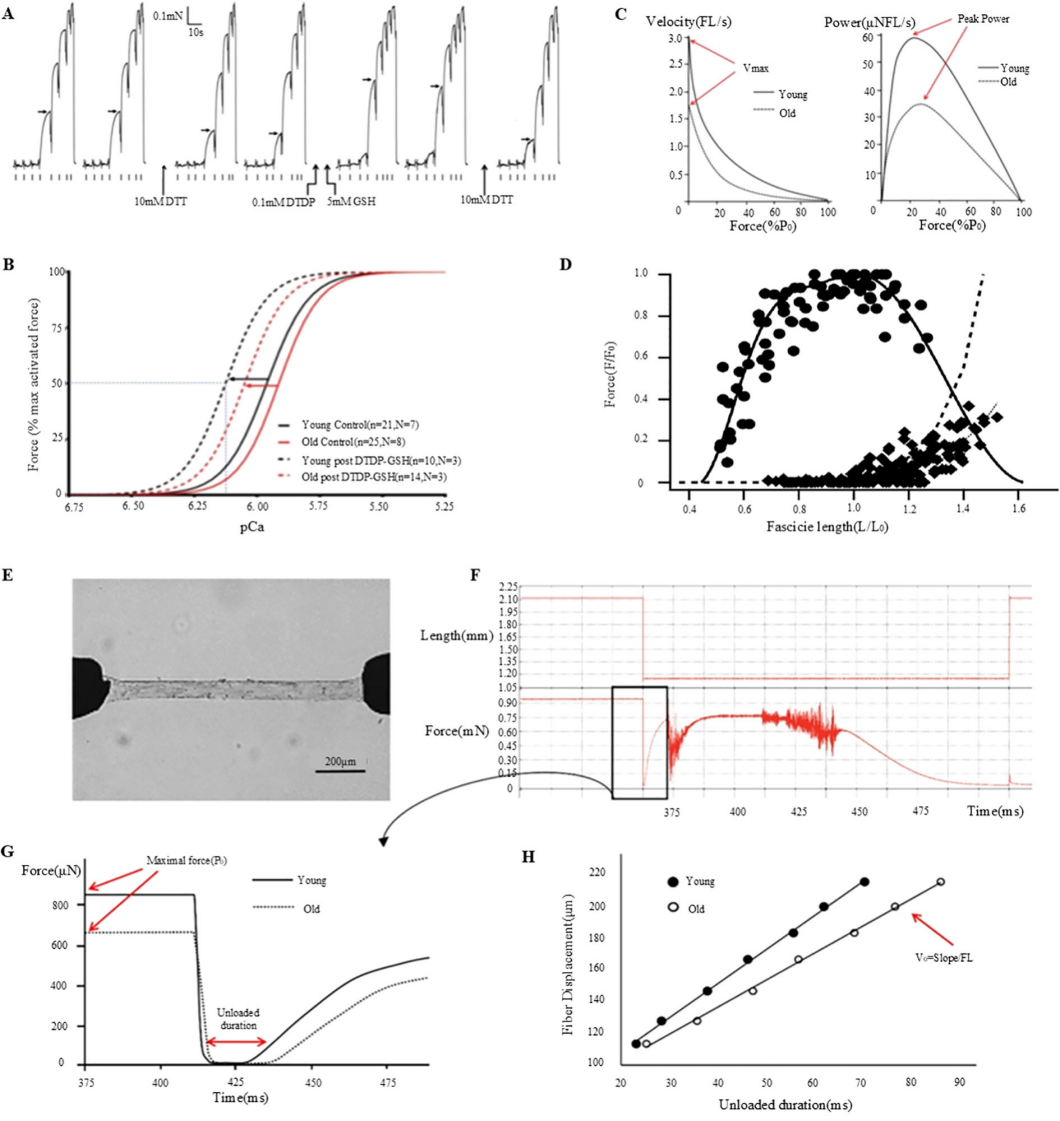
Muscle fiber mechanics and function: effects of DTT, DTDP, and GSH and age-related changes. **(A)** Investigation of the effects of DTT, DTDP, and GSH on the calcium sensitivity of the contractile apparatus in type II lateral femoral muscle fibers in elderly individuals (80). **(B)** Analysis of pCa50 and Hill coefficient values derived from Hill curves, based on the mean of individual fit values to the force-pCa relationship, plotted before (control) and after S-glutathionylation treatment for each type II muscle fiber in both young and elderly subjects (80). **(C)** Examination of the relationship between muscle velocity and force during isotonic contraction (81). **(D)** Assessment of the *in vivo* force-length relationship of the human soleus muscle (82). **(E)** Description of the experimental setup, which includes permeabilized fibers connected to a force transducer and servomotor (81). **(F)** Outline of the experimental procedure involving the transfer of fibers to an activation solution for relaxation testing (81). **(G)** Comparative analysis of the maximum force (Po) and no-load duration in type-I fibers of young and elderly subjects (81). **(H)** Quantification of the no-load duration across various relaxation lengths (81).

Although macro-assessment techniques can provide important parameters during testing, they do not sufficiently capture more complex changes within the muscles. To address these limitations, microscopic assessment methods such as dermatomechanical fiber testing have been proposed to obtain a more comprehensive understanding of muscle function (78, 79). To investigate the relationship between muscle fiber contraction force and calcium ion concentration, desmosomal fibers were subjected to a series of solutions with varying calcium ion concentrations (**Figure 3A**) (83). The collected data were subsequently fitted to Hill equations using GraphPad Prism software to calculate the calcium ion concentration (pCa50) and Hill coefficient (h) that produced the half-maximal force (**Figure 3B**) (83). The characteristic force-velocity and force-power curves for skeletal muscle behavior are presented in **Figure 3C** (84). PCa50 represents the negative logarithm of the calcium ion concentration required to elicit a half-maximal force, whereas the Hill coefficient reflects the steepness of the curve, indicating muscle sensitivity to changes in calcium ions. By analyzing the pCa50 and Hill coefficients to evaluate the mechanical properties of muscle fibers, these tests provided key insights into the specific effects of chemotherapy on muscle fiber characteristics. Various muscle function assessment techniques and their applications are shown in **Table 1**.

**Table 1:** Principles, advantages and disadvantages of muscle assessment techniques.

Technology	Principle	Advantages	Limitations	References
Handheld Dynamometer	Measures force generated during maximal voluntary contraction	Portable, easy to use	Cannot capture complex internal muscle changes	(85)
Hydraulic Dynamometer	Specifically designed to assess forearm and hand muscle strength	High accuracy, reliable	Heavy, not easily portable	(80)
Skin Mechanical Fiber Testing	Stimulates muscle fibers using solutions with varying calcium ion concentrations	Directly reflects muscle contraction characteristics	Complex technique, requires specialized equipment	(86)
Dual-Energy X-ray Absorptiometry (DXA)	Measures muscle mass using X-ray absorption differences	High accuracy, comprehensive	Radiation exposure, high cost	(87)
Computed Tomography (CT)	Evaluates muscle volume and density using X-ray imaging	High resolution, detailed images	Radiation exposure, high cost	(88)
Magnetic Resonance Imaging (MRI)	Uses magnetic fields and radio waves to image muscle structures	No radiation, high soft tissue contrast	High cost, not suitable for patients with metal implants	(89)
Bioelectrical Impedance Analysis (BIA)	Measures muscle mass by assessing electrical current flow through the body	Portable, cost-effective	Accuracy affected by various factors	(81)
Ultrasound Imaging	Uses sound waves to monitor muscle structure and function in real-time	Non-invasive, real-time monitoring	Operator-dependent	(82)
Magnetic Resonance Elastography (MRE)	Combines MRI and vibration technology to assess muscle stiffness and elasticity	High precision, non-invasive	Expensive equipment, complex technology	(90)
Electrical Impedance Myography (EIM)	Measures muscle electrical impedance properties at different frequencies	Portable, cost-effective	Limited specificity, affected by external factors	(91)

### 3.2 Evaluation of muscle mass

Traditional methods for assessing muscle mass primarily rely on imaging techniques that provide comprehensive information about muscle volume and density (84, 92). For instance, the Dual-Energy X-ray Absorptiometry (DXA) is a commonly used non-invasive approach for quantifying whole-body and regional muscle mass (84, 87). This technique can potentially differentiate muscle, fat, and bone tissues by contrasting X-ray absorption at various energy levels. DXA scans specifically measure muscle mass in appendages serving as important diagnostic indicators of sarcopenia (84, 93). The advantages of DXA include ease of administration, rapid scanning, and capability to capture muscle mass data from multiple bodily sites. Alternatively, Computed Tomography (CT) and Magnetic Resonance Imaging (MRI) offer higher resolution assessments of muscle mass, providing detailed insights into muscle anatomy and composition (94). These modalities facilitate the analysis of localized muscle alterations, including muscle atrophy or fat infiltration. In addition, CT and MRI provide precise and high-resolution imaging data of muscle tissue. Bioelectrical Impedance Analysis (BIA) is used to quantify muscle mass by assessing the passage of electrical currents through the human body (95). In principle, it works on the fact that muscle and fat tissues exhibit conductivities distinct from those of electrical currents. Although BIA is easy to administer and is cost-effective, its accuracy is significantly influenced by the hydration status and operator proficiency. Although macro-assessment techniques provide a rapid overall evaluation during testing, they do not effectively capture deeper muscular alterations. To address these limitations, microscopic assessment techniques that utilize isolated permeable single-muscle fiber samples to examine the fundamental mechanical attributes of skeletal muscles have been proposed (96). Permeability fibers offer direct insights into the functionality of contractile proteins (such as myosin, actin, and other myosins) when activated at peak calcium concentrations (97). Measurements such as the maximum force (Po), specific force (force adjusted for cross-sectional area), and no-load shortening velocity (Vo) indicate the active mechanical properties of single muscle fibers. In **Figure 3D**, the essential procedures and measurements acquired through this method are detailed (84). **Figure 3E** depicts the experimental setup of the permeabilized fibers connected to a force sensor and servomotor (84). **Figure 3F** illustrates the process of transferring the fibers to an activation solution for relaxation testing (84). **Figure 3G** compares the maximum force (Po) and no-load duration (the time required for tension to re-establish itself) for young and old Type I fibers (84). **Figure 3H** shows the no-load durations measured at different relaxation lengths from which the no-load shortening velocity (Vo) was calculated (84).

Therefore, macro-level techniques for assessing muscle mass are advantageous for preliminary evaluation whereas microscopic methods provide a more comprehensive understanding of muscle functions and characteristics. This has been confirmed by studies investigating the effects of sarcopenia and aging on skeletal muscles, where microscopic assessments have demonstrated superior efficacy (84).

Integrating macro- and micro-measurements can help researcher obtain a more comprehensive understanding of muscle function across diverse movement patterns. Specifically, combining alterations in muscle fiber length, as quantified through ultrasound imaging, with joint kinematic data obtained via motion capture technology, can effectively reveal muscle function during specific movements, making it ideal for application in exercise training, rehabilitation therapy, and biomechanical research. The comprehensive nature of these measurements not only enhances our understanding of the muscular function in various athletic and daily activities, but also provides important ideas for improving muscle performance through targeted training or therapeutic interventions (**Figure 3D**) (84). This holistic methodology provides insights into the intricacies of muscle function and the scope of applications (84).

### 3.3 Evaluation of muscle function

**Figure 4:**
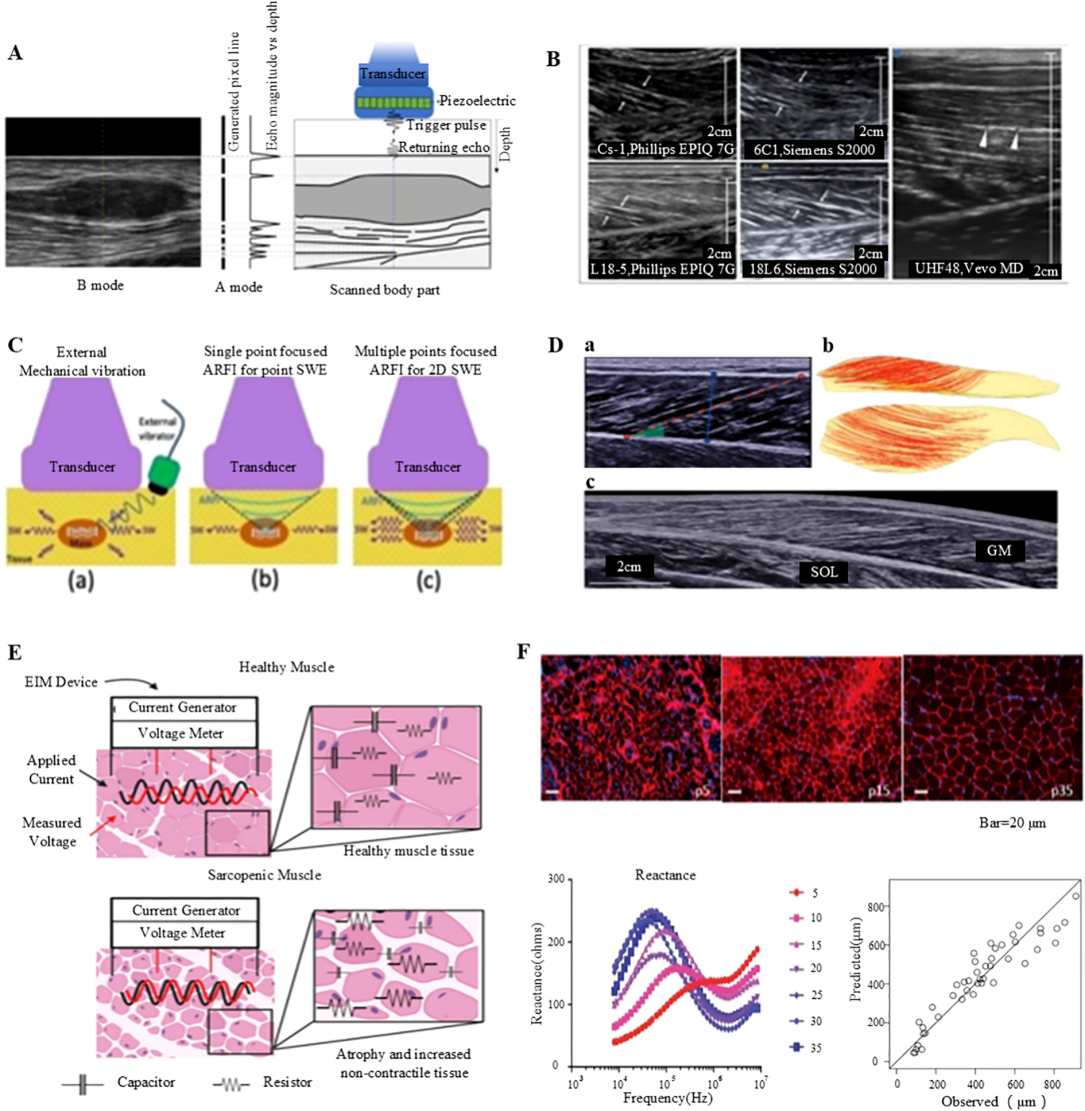
Non-invasive assessment of muscle function: ultrasound imaging and electrical impedance myography. **(A)** Generation of A- and B-mode ultrasonography (98). **(B)** Longitudinal B-mode ultrasound imaging of the medial gastrocnemius muscle in a healthy volunteer (98). **(C)** B-mode ultrasound imaging of the gastrocnemius muscle conducted with a linear transducer (98). **(D)** Comprehensive analysis of the medial gastrocnemius muscle (GM) utilizing ultrasonography: **(a)** B-mode ultrasound imaging performed with a linear transducer, **(b)** Diffusion tensor imaging (DTI) fiber reconstruction of the GM, and **(c)** Extended field-of-view ultrasound imaging of the GM (99). **(E)** Conceptual illustration of impedance measurements in healthy versus sarcopenic muscles, highlighting increased non-contractile tissue and reduced myocyte size (100). **(F)** Illustration of employing electrical impedance myography (EIM) data to predict muscle fiber size (100).

Ultrasound imaging is a non-invasive, real-time assessment tool that is extensively utilized to assess muscle function (98, 99). This imaging technique offers various modes for evaluating muscle structure and function, including A-mode, B-mode, M-mode, and Doppler mode. The A-mode or amplitude mode is a pioneering ultrasound imaging technique (17, 98, 99). It measures and displays the intensity of reflections from tissue interfaces using a single beam of sound (**Figure 4A**) (99). Although it is primarily to assess tissue thickness and locate reflective interfaces, A-mode ultrasound, although less commonly applied in clinical practice, it is used in specific scenarios. On the other hand, the B-mode, or Brightness mode, ultrasound imaging is the most frequently used modality (101). It can display muscle structure and morphology by generating grayscale images. In this mode, ultrasound waves are reflected as they pass through tissues, producing distinct gray values that correspond to the tissue density and structural characteristics (**Figures 4B, D**) (17, 99). M-mode, or Motion mode, ultrasound imaging is utilized to assess muscle activity during movement (17, 98, 99). It captures and displays the real-time trajectory of muscle motion, making it particularly suitable for cardiac ultrasound and for evaluating muscle contractile function. Doppler-mode ultrasound imaging can assess blood flow by quantifying the shift in the frequency of the ultrasonic waves (102). This technique allows clinicians to identify and measure blood flow velocity and direction, providing important details regarding muscle blood supply and detecting vascular abnormalities. In ultrasound images, normal muscle tissue exhibits a homogeneous structure with moderate echogenicity, well-aligned fibers, and distinct continuous reflections of myofascial and fascial tissues (103). Any abnormalities such as muscle atrophy, fibrosis, or inflammation can be detected based on variations in echo characteristics (104).

The MRI technique is recognized as the definitive method for evaluating skeletal muscle mass and composition (68). This imaging modality provides highly precise and reproducible analysis of muscle volume and fat infiltration. The primary applications of MRI in skeletal muscle research are as follows.

#### 3.3.1 Structural imaging

MRI generates high-resolution images of muscle structures, providing detailed visualization of muscle fiber arrangement and tissue status (68).

#### 3.3.2 Magnetic resonance elastography

MRE uses MRI data to assess the tissue elasticity. Through the application of vibrations and measurement of the resultant displacements, MRE estimates the shear wave velocity and elasticity of the tissue, facilitating assessment of the muscle stiffness and elasticity (68).

Elastography is a technique employed to evaluate tissue stiffness and elasticity, classified into two main types. Quasi-static elastography measures tissue deformation in response to externally applied pressure, making it suitable for assessing the stiffness of superficial tissues (105). Conversely, dynamic elastography utilizes acoustic waves to determine the shear wave velocity within a tissue, and by analyzing this wave velocity, the tissue’s modulus of elasticity can be obtained (106). This method provides a more precise assessment of the elastic properties of the deeper tissues. Elastography has several applications in skeletal muscle research, including evaluation of muscle stiffness, modulus of elasticity, and muscle damage (99, 106). In a previous study, shear wave elastography was employed to assess stiffness changes in rotator cuff tendinopathy, demonstrating a strong correlation with the MRI findings (**Figure 4C**) (99).

Electrical Impedance Myography (EIM) is a non-invasive assessment technique used to examine the muscle tissue structure and function based on the electrical impedance (107). It allows high-resolution and portable monitoring of muscle mass, adipose tissue content, and overall muscle health. In clinical practice, ultrasound imaging and EIM are the common modalities for assessing muscle function which is principally due to their non-invasive nature, real-time capabilities, and high-resolution data (100). The EIM technology employs parameters, such as resistance, reactance, and phase angle, vary with the frequency of the input current, providing important insights into the structure and integrity of the muscle (**Figure 4E**) (100). For instance, the EIM measurements of muscle fiber atrophy indicate increased resistance and decreased reactance values within a specific frequency range, accompanied with elevated fat infiltration which reduce resistance and decreases anisotropy ratios (108). Moreover, high glycogen accumulation and vacuole formation elevate both resistance and reactance values at high frequencies. Despite its numerous advantages, the EIM has certain limitations, including the need for highly standardized measurement techniques and it is mainly applicable for superficial muscle measurements. Thus, further technical advancements are necessary to optimize its performance in deeper muscles (**Figure 4F**) (100). Notwithstanding these limitations, the EIM is considered a robust tool for evaluating skeletal muscle health, owing to its rapid, cost-effective, portable, and user-friendly properties.

## 4 Impact of immunotherapy and immune microenvironment in tumor treatment

### 4.1 Application of immune checkpoint inhibitors

**Figure 5:**
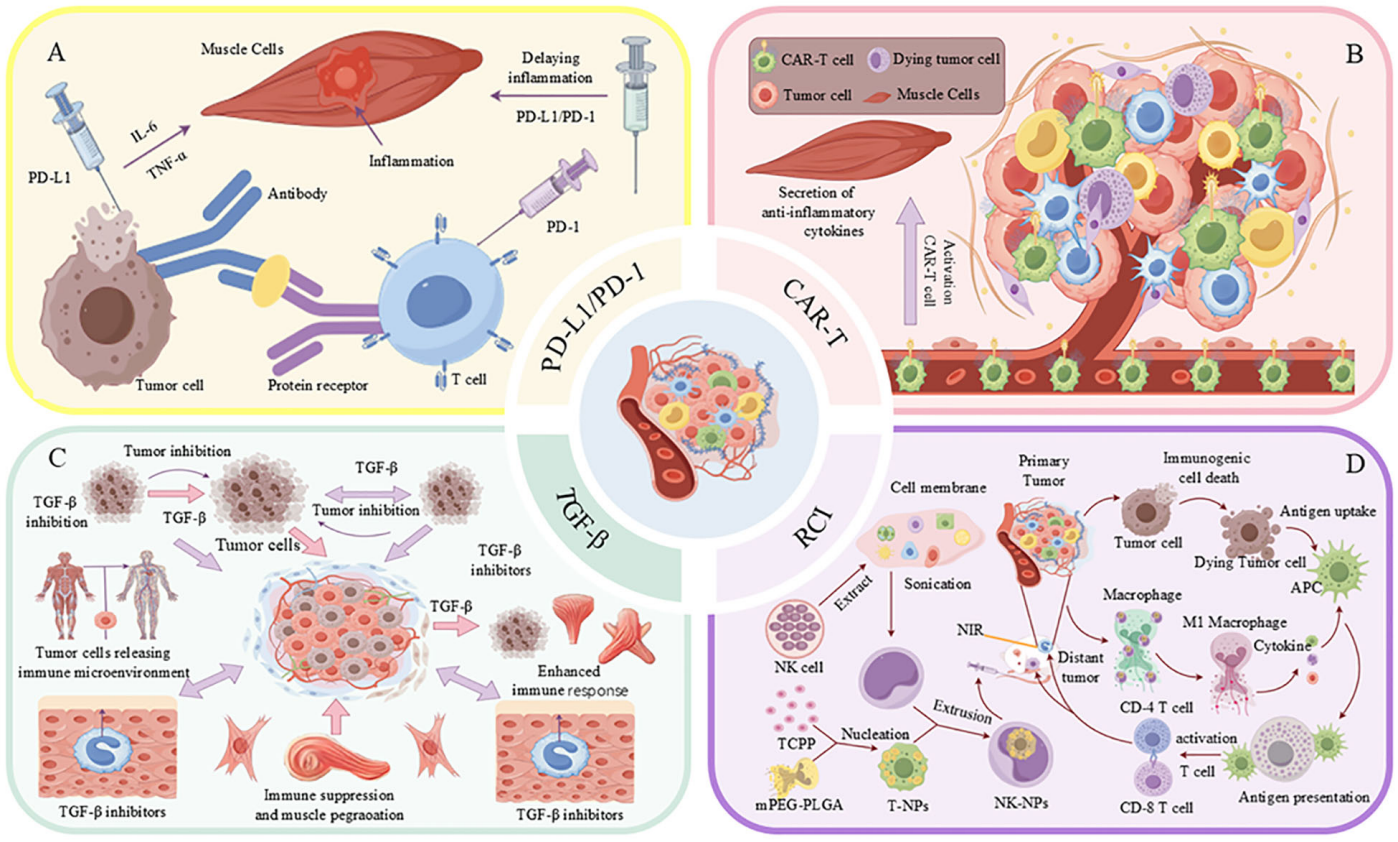
Strategies for combined treatment of tumors with immunotherapy and the TME. **(A)** Tumor cells impede the immune response through the interaction of PD-L1 with PD-1 on T-cells, while simultaneously inducing inflammation via the secretion of TNF-α and IL-6. **(B)** CAR-T cells contribute to the tumor microenvironment (TME) by secreting anti-inflammatory cytokines and activating M1-type macrophages. **(C)** Inhibition of TGF-β enhances the immune response and suppresses tumor growth. **(D)** The activation of immune cells through the application of nanoparticles, such as T-NPs and NK-NPs, in conjunction with near-infrared (NIR) light irradiation, facilitates anti-tumor immune responses.

Programmed cell death protein 1 (PD-1) and its ligand, programmed death-ligand 1 (PD-L1), serve as pivotal immune checkpoints that regulate T cell immune responses (109). Tumor cells can circumvent immune detection by upregulating PD-L1 expression, which interacts with PD-1 on T cells, thereby suppressing T cell proliferation and effector functions (110). Inhibitors targeting the PD-1/PD-L1 axis disrupt this interaction, thereby restoring T cell activity and augmenting anti-tumor immune responses (**Figure 5A**). These immune checkpoint inhibitors not only induce apoptosis in tumor cells but also contribute to the modulation of immune homeostasis, suppression of chronic inflammation, and improvement of patient survival rates (111). In addition to their direct anti-tumor effects, PD-1/PD-L1 inhibitors have been shown to mitigate cancer-associated cachexia, a syndrome marked by significant muscle and fat loss that adversely affects quality of life and treatment tolerance (83, 112). These inhibitors achieve this by reducing the secretion of pro-inflammatory mediators and diminishing the activity of immunosuppressive cells, such as regulatory T cells and M2-type macrophages, thereby alleviating muscle atrophy (22). PD-1/PD-L1 inhibitors contribute to the improvement of the TME by modulating the metabolic activities of tumor-associated macrophages and enhancing the supply of oxygen and nutrients (22, 113). Specifically, these inhibitors can induce the polarization of macrophages from the M2 phenotype, which is associated with tumor promotion, to the M1 phenotype, which exhibits anti-tumor properties, thereby reducing immune suppression and mitigating muscle wasting (114). Furthermore, PD-1/PD-L1 inhibitors have the potential to preserve skeletal muscle mass by enhancing nutrient metabolism and promoting protein synthesis in patients with tumors (115). Evidence suggests that inhibition of the PD-1/PD-L1 pathway can suppress muscle protein degradation pathways, including the ubiquitin-proteasome system and autophagy-lysosome systems, thus preventing muscle loss (116). Recent studies show that PD-1/PD-L1 inhibitors improve skeletal muscle function. A retrospective study found a 20% reduction in muscle wasting in non-small cell lung cancer patients treated with PD-1 inhibitors compared to those on conventional chemotherapy (117). Additionally, a prospective study reported improved muscle strength and a 30% decrease in severe muscle weakness in melanoma patients using these inhibitors. These benefits, which include anti-inflammatory effects and enhanced nutrient metabolism, suggest that PD-1/PD-L1 inhibitors help maintain muscle integrity and function (117, 118). Continued research is essential to further elucidate these mechanisms and optimize the therapeutic application of PD-1/PD-L1 inhibitors, potentially expanding their role in cancer treatment.

CTLA-4 inhibitors play a pivotal role in cancer immunotherapy by activating effector T cells to mitigate tumor-induced immunosuppression (119). These inhibitors function by binding to CTLA-4 on T cells, thereby preventing its interaction with CD80 and CD86 on antigen-presenting cells. This blockade facilitates the restoration of T cell activation and proliferation, thereby augmenting the anti-tumor immune response (120). Additionally, CTLA-4 inhibitors have the capacity to selectively deplete tumor-infiltrating regulatory T cells, a process mediated by macrophages with Fcγ receptors within the TME. Their efficacy in melanoma treatment is partly attributed to the elimination of Tregs (119). Clinical evidence substantiates the beneficial impact of CTLA-4 inhibitors on skeletal muscle function. A prospective study involving melanoma patients treated with ipilimumab, a CTLA-4 inhibitor, reported a 25% enhancement in muscle strength and a 15% reduction in symptoms of muscle wasting compared to those receiving standard therapy (120, 121). Additionally, a retrospective analysis of patients with advanced renal cell carcinoma indicated that individuals treated with CTLA-4 inhibitors exhibited decreased levels of pro-inflammatory cytokines, such as IL-6 and TNF-α, alongside improved muscle mass, as assessed by DXA (122). These findings imply that CTLA-4 inhibitors not only augment anti-tumor immunity but also ameliorate muscle wasting by attenuating systemic inflammation and enhancing metabolic homeostasis. The underlying mechanisms of these benefits include the depletion of immunosuppressive regulatory Tregs and the restoration of balanced immune responses, which collectively contribute to the preservation of muscle integrity and function (123). Furthermore, these inhibitors demonstrate potential in mitigating muscle wasting induced by inflammatory factors present in the TME (124). This modulation of the immune system not only addresses excessive immune reactions but also maintains immune tolerance. In summary, CTLA-4 inhibitors enhance anti-tumor effects and provide novel strategies for managing muscle wasting in cancer therapy.

### 4.2 Association of CAR-T cell therapy with the treatment of tumor-induced muscle damage

CAR-T therapy demonstrates significant efficacy in the treatment of hematological malignancies; however, its application in solid tumors is impeded by the immunosuppressive TME (125, 126). This TME not only obstructs CAR-T cell functionality but also exacerbates cancer-related cachexia, a leading cause of mortality among patients with solid tumors (127). Pro-inflammatory cytokines, such as IL-6, TGF-β, and TNF-α, present within the TME, are implicated in the induction of muscle atrophy and dysfunction. Clinical evidence suggests that CAR-T cell therapy may inadvertently exacerbate muscle wasting in patients with solid tumors. A retrospective analysis of individuals with advanced colorectal cancer undergoing CAR-T therapy reported a 30% incidence of muscle weakness and atrophy, accompanied by elevated levels of pro-inflammatory cytokines, including IL-6 and TNF-α (128). In contrast, a preclinical study indicated that modifying CAR-T cells to secrete anti-inflammatory cytokines, such as IL-10, reduced muscle inflammation and preserved muscle function in murine models. These findings underscore the dual impact of CAR-T therapy on skeletal muscle, highlighting the necessity for strategies that mitigate muscle toxicity while enhancing anti-tumor efficacy (129). To enhance the effectiveness of CAR-T therapy and mitigate muscle damage, strategies have been proposed, including the engineering of CAR-T cells for prolonged persistence and the secretion of anti-inflammatory cytokines (**Figure 5B**), as well as the combination of CAR-T therapy with IL-6 inhibitors, TGF-β blockers, or metabolic regulators. Preclinical investigations indicate that targeting TGF-β can alleviate muscle wasting and enhance the anti-tumor efficacy of CAR-T cells (130–132). Current research endeavors focus on optimizing these strategies to improve both oncological outcomes and muscle health, thereby potentially enhancing patient survival and quality of life (133).

### 4.3 Modulation of the tumour microenvironment to improve muscle function

#### 4.3.1 Anti-TGF-β therapy

The TGF-β is a major cytokine involved in the regulation of various biological processes, including cell growth, differentiation, apoptosis and immune regulation (134). However, in the TME, high TGF-β expression promotes tumor progression, immunosuppression, and treatment resistance (135). Studies have shown that TGF-β not only promotes tumor cell growth and metastasis, but also inhibits the body’s immune surveillance function via multiple mechanisms, allowing tumor cells to evade immune attack (136). TGF-β promotes the expansion and accumulation of immunosuppressive cells (e.g., regulatory T cells, Tregs, and myeloid-derived suppressor cells, MDSCs), leading to the suppression of anti-tumor activity of effector T cells (137). This immunosuppressive effect reduces the ability of the immune system to eliminate tumor cells (**Figure 5C**). In addition, TGF-β can activate cancer-associated fibroblasts and induce extracellular matrix remodeling, leading to establishment of a conducive environment that favors tumor growth while preventing immune cell infiltration (138). Studies have shown that TGF-β can promote angiogenesis and increase the blood supply to tumor tissues, generating more oxygen and nutrients needed for tumor growth and metastasis. In addition, TGF-β increases the viability of tumor cells by regulating their metabolic pathways and promoting immune escape by altering energy metabolism.

Considering that TGF-β is an important component of the tumour microenvironment, its inhibition can be a promising therapeutic strategy. Inhibition of TGF-β improves the efficacy of immune checkpoint inhibitors, and overexpression of TGF-β expression causes tolerance to immune checkpoint inhibitors (for example, PD-1/PD-L1 and CTLA-4 antibodies), and that inhibition of TGF-β can reverse this tolerance, thereby increasing the response rate to immunotherapy (139). In addition, TGF-β inhibition enhances the Tregs and MDSCs accumulation as well as the anti-tumor activity of CD8+ T cells, resulting in enhanced TME and improved immune response (140). TGF-β inhibition also reduces the cancer cells invasiveness and metastatic potential to distant organs. When combined with other therapeutic approaches (e.g., radiotherapy, chemotherapy, and targeted therapies), TGF-β inhibitors exhibit synergistic effects, improving the overall therapeutic effect (141). Besides its role in cancer therapy, TGF-β inhibition can potentially address cancer-related muscle atrophy (cancer cachexia). In cancer patients, TGF-β was found to prevent loss of muscle mass and function by accelerating the degradation of muscle proteins through activation of the ubiquitin-proteasome pathway (UPS) and autophagy-lysosomal pathways. TGF-β inhibition suppressed the signalling of the pathways, slowing down muscle loss. Overexpression of TGF-β inhibits the proliferation and differentiation of muscle stem cells (satellite cells), decreasing their capacity for repair. TGF-β inhibition inhibits satellite cells and promotes muscle regeneration and repair. Elevated TGF-β expression was reported to be associated with chronic inflammation, which exacerbated muscle wasting (142). Other studies have reported that TGF-β inhibition can reduce the secretion of pro-inflammatory factors in the muscles, improving muscle function. TGF-β inhibition not only enhanced the anti-tumor immune response but also indirectly improved muscle health, improve the physical status and quality of life for patients. Therefore, high expression of TGF-β in the TME not only promotes cancer progression but also exacerbates muscle wasting. These findings indicate that anti-TGF-β therapy can treat cancer as well as cancer-related muscle atrophy. A combination of immunotherapy, chemotherapy, or targeted therapy, TGF-β inhibitors can provide better treatment effects, while preventing the decline in muscle function. Further clinical studies are needed to optimize TGF-β inhibition strategies to promote personalized cancer treatment. Besides its use in cancer therapy, TGF-β inhibition can potentially improve cancer-related muscle wasting (cancer cachexia). Patients with cancer often experience loss of muscle mass and function, and TGF-β plays an important role in this process (142). TGF-β has been shown to accelerate the degradation of muscle proteins through the activation of the UPS and autophagy-lysosomal pathways, ultimately resulting in muscle atrophy. Inhibiting TGF-β activity mitigates the activation of these pathways, thereby decelerating muscle loss. Furthermore, research indicates that elevated levels of TGF-β suppress the proliferation and differentiation of muscle stem cells, known as satellite cells, which impairs the muscle repair process. The inhibition of TGF-β can restore the functionality of satellite cells, thereby facilitating muscle regeneration and repair. Additionally, high TGF-β expression is frequently correlated with chronic inflammation, which further exacerbates muscle wasting. By inhibiting TGF-β, the levels of pro-inflammatory factors within the muscle can be reduced, leading to improved muscle function. Notably, TGF-β inhibition not only enhances the anti-tumor immune response but also indirectly promotes muscle health, thereby enabling patients to maintain a better physical condition and quality of life. Moreover, elevated TGF-β expression within the TME not only facilitates cancer progression but also contributes to muscle wasting. Consequently, anti-TGF-β therapy emerges as a significant approach in cancer treatment and may represent a novel strategy for improving cancer-related muscle deterioration.

#### 4.3.2 Targeting the muscle-tumor connection

Tumors negatively impact muscle function through the secretion of various factors, notably TNF-α, which contributes to muscle atrophy and dysfunction (143). TNF-α is a key pro-inflammatory cytokine implicated in muscle degradation across a range of diseases. In the context of cancer cachexia, elevated TNF-α levels facilitate muscle protein degradation and the consequent loss of muscle mass. Furthermore, TNF-α exacerbates muscle degradation by interacting with its receptor and activating several downstream signaling pathways, including the NF-κB pathway (144). Pharmacological interventions targeting TNF-α and its signaling pathways, such as TNF inhibitors, have demonstrated potential in delaying muscle wasting in preclinical studies. These agents mitigate the inflammatory response by inhibiting TNF-α activity, thereby decelerating muscle protein breakdown and the progression of muscle atrophy. Nonetheless, despite promising outcomes in animal models, the clinical efficacy of TNF inhibitors remains inconsistent, potentially due to individual variability and the complexity of the disease (145). Other cytokines, including interleukin-6 (IL-6), play a significant role in muscle wasting. Empirical evidence suggests that IL-6 exacerbates muscle wasting by exerting deleterious effects on muscle tissue. In addition to TNF-α inhibitors, alternative anti-inflammatory strategies, such as IL-6 inhibitors and TGF-β blockers, present promising approaches for alleviating muscle wasting. IL-6, a pro-inflammatory cytokine, has been demonstrated to contribute to muscle atrophy by facilitating protein degradation and hindering muscle regeneration (146). Transforming growth factor-beta (TGF-β), recognized for its involvement in immune evasion and fibrosis, also promotes muscle wasting by enhancing the activity of proteasome systems (147). Targeting these cytokines may reduce muscle protein degradation and enhance muscle function. In conclusion, a comprehensive approach is essential for addressing the interplay between muscle and tumor, which involves targeting pro-inflammatory cytokines such as TNF-α, IL-6, and TGF-β, in conjunction with nutritional interventions aimed at supporting muscle health. Future research should prioritize the optimization of these strategies to enhance clinical efficacy and improve patient outcomes.

### 4.4 Combination therapy

#### 4.4.1 Radiotherapy with immunomodulators

The mechanism of action of radiotherapy in cancer treatment extends beyond the direct cytotoxic effects on tumor cells, incorporating the enhancement of the immune response through the release of tumor antigens (148) This immunostimulatory effect primarily arises from radiotherapy-induced immunogenic cell death (ICD), which is marked by the emission of damage-associated molecular patterns (DAMPs) and tumor antigens. These elements are capable of activating both innate and adaptive immune responses, thereby facilitating the immune system’s recognition and elimination of tumor cells. Furthermore, radiotherapy can augment the anti-tumor immune response by modifying the TME, promoting the infiltration and activation of immune cells (149). Combining radiotherapy with immunomodulators can further mitigate tumor- and treatment-induced systemic inflammation and muscle damage. For instance, the integration of radiotherapy with immune checkpoint inhibitors may produce a synergistic effect, enhancing the anti-tumor immune response and potentially improving clinical treatment outcomes (150). This combination therapy not only facilitates the immunogenic demise of tumor cells but also augments their capacity to identify and eradicate tumors through the activation of immune cells, including T cells and NK cells. Moreover, radiotherapy can bolster tumor-specific immunity by eliciting an acute inflammatory response, thereby enhancing therapeutic efficacy. As illustrated in **Figure 5D**, radiotherapy can potentiate the immune system via near-infrared (NIR) light irradiation by activating nanoparticles that encapsulate components derived from tumor cell membranes or NK cell membranes, which mimic tumor antigens and further stimulate the immune response (151). These nanoparticles amplify the immune system’s assault on tumors by promoting the activation of immune cells and inflammatory responses within the TME.

Radiotherapy also promotes the polarization of macrophages from the immunosuppressive M2 type to the anti-tumor M1 type, thereby enhancing the anti-tumor immune response in the TME (152). In conclusion, radiotherapy not only controls tumors through direct cytotoxic effects but also enhances systemic anti-tumor immune responses through complex immunomodulatory mechanisms. The understanding and application of such mechanisms provide a theoretical basis for the combination of radiotherapy and immunotherapy, which may offer new hope for cancer treatment. By studying the interaction between radiotherapy and immunomodulators in detail, we can develop more effective cancer treatment protocols that not only control the growth and spread of tumors but also improve the quality of life and prognosis of patients.

#### 4.4.2 Integrating immunotherapy with nutrition

Malnutrition and tumor-induced metabolic disorders are significant factors contributing to the decline in muscle function among cancer patients. Cancer cachexia, marked by ongoing muscle degeneration and dysfunction, is a common and debilitating condition (153). Studies have emphasized the impact of inflammation and oxidative stress in disrupting the pathways of muscle protein synthesis and degradation, resulting in muscle wasting (154). In response to this issue, nutritional interventions are increasingly being recognized for their potential to mitigate these adverse effects. High-protein diets play a critical role in supplying the amino acids required for muscle protein synthesis, thereby mitigating the catabolic effects associated with cancer and its treatment. Certain amino acids, notably leucine, have been demonstrated to activate the mTOR pathway, which is vital for facilitating muscle growth and preventing atrophy (155). Additionally, omega-3 fatty acids, present in fish oil, enhance this strategy by diminishing the production of pro-inflammatory cytokines such as TNF-α and IL-6, which are involved in muscle protein degradation. Research suggests that omega-3 supplementation can enhance muscle function and alleviate fatigue in cancer patients undergoing immunotherapy (156). The integration of immunotherapy with specific nutritional strategies presents a synergistic approach to improving treatment outcomes. For instance, the combination of immune checkpoint inhibitors with high-protein diets and omega-3 fatty acids has the potential to enhance anti-tumor immune responses and promote muscle health (157). This approach not only fortifies the immune system but also mitigates the inflammatory burden associated with muscle wasting.

Additional anti-inflammatory supplements, such as curcumin and green tea extract, may contribute to improved muscle health by inhibiting NF-κB signaling, thereby mitigating inflammation and oxidative stress in muscle tissue (158). Considering the diverse nutritional status and metabolic requirements of cancer patients, it is essential to develop individualized nutritional plans based on metabolic profiling and inflammatory markers to optimize treatment efficacy and minimize adverse effects (157). Future research should focus on examining the long-term effects of integrating immunotherapy with nutritional interventions. Clinical trials are necessary to establish standardized protocols for nutritional support in cancer patients undergoing immunotherapy. Investigating the role of gut microbiota in modulating immune responses and muscle health offers a novel avenue for intervention (157). In conclusion, the integration of immunotherapy with nutritional interventions represents a promising strategy for enhancing treatment efficacy and improving patient quality of life. By addressing both the tumor and its effects on muscle health, these combined approaches aim to achieve improved clinical outcomes in cancer management.

## 5 Challenges and future directions

In recent years, immunotherapy has made remarkable progress in the treatment of tumors, particularly in the regulation of the TME. However, this field still faces several challenges that limit its widespread application and therapeutic effects. Moreover, the impact of immunotherapy on skeletal muscle and its potential role in muscle preservation or damage during cancer treatment remain underexplored, necessitating further investigation (75).

First, the complexity and diversity of the TME severely restrict the efficacy of immunotherapy. The TME contains a variety of cellular components, cytokines, blood vessels, and stroma that interact to create an environment that supports tumor growth and metastasis while suppressing immune responses (159). For example, hypoxia is a prominent feature of the TME, and studies have shown that it not only affects the metabolism and growth of tumor cells but also facilitates immune escape, which severely weakens the efficacy of immunotherapy. A hypoxic environment enhances tumor immune evasion by promoting the accumulation of immunosuppressive cells (e.g., Tregs and MDSCs) and inducing the expression of immune checkpoints. Therefore, modulation of the hypoxic microenvironment has become an important strategy for improving immunotherapy efficacy. Additionally, recent evidence suggests that hypoxia and inflammation in the TME may also contribute to muscle wasting in cancer patients (159). Chronic systemic inflammation and immune dysregulation associated with immunotherapy could exacerbate skeletal muscle atrophy, further impairing patients’ quality of life and treatment outcomes.

The introduction of nanotechnology offers new solutions to address these challenges. Nanomaterials have unique physicochemical properties that allow them to play a crucial role in targeted drug delivery and TME modulation. They can enhance the efficacy of immunotherapy by improving drug targeting and penetration (160). In particular, nanomaterials can improve the response rate of immunotherapy by penetrating tumor tissues, targeting tumor cells, and reducing immunosuppressive factors in the TME. Additionally, nanotechnology can be used to carry immunomodulators, such as immune activators, cytokines, or immune checkpoint inhibitors, which directly enhance immune cell function and enable the immune system to recognize and attack tumor cells more effectively. These advances highlight the great potential of nanotechnology in improving immunotherapy, particularly for cancer treatment (70). However, the effects of nanotechnology-based immunotherapy on skeletal muscle remain unclear. Given that systemic immune activation can lead to muscle inflammation and oxidative stress, nanomaterials must be carefully designed to minimize unintended adverse effects on muscle tissue while maintaining their anti-tumor efficacy.

Despite progress in immunotherapy and TME modulation, many pressing issues still need to be addressed. One major challenge is the effective combination of immunotherapy with other therapeutic approaches (e.g., chemotherapy, radiotherapy, or targeted therapies) to achieve a more integrated treatment effect. Combining different therapeutic strategies can exploit their respective strengths, overcome the limitations of single therapies, and improve treatment efficacy and tolerability (70). Additionally, immune escape and drug resistance remain significant hurdles. Tumor cells evade immune surveillance by constantly altering surface markers and inducing an immunosuppressive microenvironment, which not only leads to immunotherapy failure but also contributes to tumor recurrence and metastasis. Overcoming these immune escape mechanisms and restoring the anti-tumor function of the immune system remains a critical area of current research. Moreover, cancer-induced muscle loss, or cachexia, remains a major challenge in cancer treatment. Immunotherapy-induced cytokine release may exacerbate muscle protein degradation pathways, leading to further muscle atrophy (161). Therefore, strategies to mitigate muscle loss, such as incorporating anti-inflammatory agents, exercise interventions, or metabolic modulators alongside immunotherapy, warrant further investigation.

In terms of emerging therapeutic approaches, CAR-T cell therapy has shown great potential. By genetically engineering patients’ T cells to express CARs, these modified T cells can specifically recognize and kill tumor cells expressing certain antigens (162). However, the application of CAR-T cell therapy in solid tumors is limited by the immunosuppressive nature of the TME. To overcome this, researchers have developed several strategies, such as genetically modifying CAR-T cells to enhance their survival and anti-tumor activity within the TME. For instance, CAR-T cells can be engineered to secrete anti-inflammatory cytokines (e.g., TGF-β), which not only boost the anti-tumor immune response but also protect muscle tissue from inflammatory damage. Additionally, combining CAR-T cell therapy with other treatments like IL-6 inhibitors, TGF-β blockers, or metabolic modulators can simultaneously improve muscle mass and function (131). Preclinical studies have demonstrated that targeting TGF-β can reduce muscle wasting and enhance the anti-tumor effect of CAR-T cells in mouse models of malignant disease. This suggests that by modulating key factors in the TME, muscle function can be protected and restored while enhancing CAR-T cell therapy efficacy.

Another emerging area is the development of novel immune checkpoint modulators. Beyond the well-studied PD-1/PD-L1 and CTLA-4 inhibitors, new inhibitors targeting other immune checkpoints such as TIM-3 and LAG-3 are under development. These novel inhibitors hold promise for overcoming resistance to existing immunotherapies and could be effective in treating different types of cancer (120). Furthermore, combining multiple immune checkpoint inhibitors has shown promising results, as blocking several inhibitory pathways at once can more comprehensively activate the immune system and enhance the intensity and durability of the anti-tumor immune response.

In terms of combination strategies, the integration of immunotherapy with anti-inflammatory treatments offers a new avenue for reducing muscle damage. Nonsteroidal anti-inflammatory drugs (NSAIDs) and selective COX-2 inhibitors can reduce muscle inflammation and injury by inhibiting inflammatory responses (163). For example, using NSAIDs in patients receiving immune checkpoint inhibitors has been shown to significantly decrease the incidence and severity of immune-related myositis. Moreover, nutritional interventions during immunotherapy have shown potential for muscle protection. Diets rich in omega-3 fatty acids and high-quality proteins can enhance muscle anti-inflammatory capacity and regenerative potential, reducing muscle atrophy. Clinical trials have indicated that such nutritional support can improve muscle strength and quality of life in patients undergoing immunotherapy.

New interventions such as gene editing technology are also being explored to optimize immunotherapy and protect muscle function (164). Using tools like CRISPR/Cas9, researchers can precisely modify immune cells to enhance their anti-tumor activity while reducing toxicity to normal tissues. For example, T cells can be edited to delete genes that may cause autoimmune reactions or to insert genes that enhance their ability to recognize and target tumors (165). Additionally, gene editing can be applied to regulate inflammatory and apoptotic pathways within muscle cells, thereby reducing muscle injury caused by immunotherapy.

The combination of immunotherapy and exercise intervention is another emerging research direction. Regular exercise can increase muscle strength and endurance, improve muscle metabolism, and reduce immunotherapy-related muscle toxicity (84). Research has shown that exercise can activate anti-inflammatory signaling pathways in muscles, reducing the production of inflammatory cytokines while promoting muscle protein synthesis and mitochondrial function recovery. This combined approach not only helps maintain and enhance muscle function but also improves patients’ tolerance and adherence to immunotherapy (84, 166).

In conclusion, the combination of immunotherapy with TME modulation holds promise for cancer treatment by enhancing therapeutic efficacy and overcoming tumor drug resistance. However, further research is needed to optimize treatment protocols and address existing technical challenges. A deeper understanding of the interplay between immunotherapy and skeletal muscle health is crucial for developing more comprehensive treatment strategies that target tumors while preserving muscle function. Looking ahead, the integration of nanotechnology, immunotherapy, and muscle-protective interventions may be a key direction for achieving breakthroughs in cancer therapy while minimizing adverse effects on muscle tissue.

## 6 Conclusion

This study investigates the mechanisms through which immunotherapy influences skeletal muscle function in cancer patients, with a particular emphasis on the interaction between immunotherapy and TME and its impact on the biological properties of skeletal muscle. While immunotherapy suppresses tumor growth by activating the immune system, it can also induce immune-related adverse effects, notably detrimental impacts on skeletal muscle (28). Specifically, immunotherapy may impair skeletal muscle function by triggering immune-associated myositis and systemic inflammatory responses, which can result in muscle mass loss and functional decline, especially in cancer patients. The TME plays a pivotal role in immunotherapy by modulating the immune response and anticancer efficacy through the regulation of immune cells and metabolic activities (167). Alterations in the TME can exacerbate skeletal muscle wasting through mechanisms such as chronic inflammation. The inflammatory response elicited by immunotherapy is closely linked to muscle wasting, and cachexia—a cancer-associated muscle wasting condition—may be more prevalent in patients undergoing immunotherapy (168). This paper also examines current biomechanical methodologies for evaluating the impact of immunotherapy on skeletal muscle and suggests strategies to mitigate the associated damage by targeting immunosuppressive cells and implementing metabolic reprogramming to enhance therapeutic efficacy. Future research should prioritize minimizing the adverse effects of immunotherapy on skeletal muscles to improve patient quality of life and clinical outcomes. In conclusion, while immunotherapy has introduced significant advancements in cancer treatment, its impact on skeletal muscle warrants greater attention. Subsequent studies should aim to enhance the therapeutic benefits of immunotherapy while mitigating its detrimental effects on skeletal muscle, thereby offering a more holistic treatment approach for cancer patients.
